# Purification, Structural Characterization, Antioxidant Activity, and *In Vitro* Digestibility of Peach Gum Polysaccharide Extracted Using an Enzymatic Method

**DOI:** 10.1002/fsn3.70545

**Published:** 2025-06-29

**Authors:** Xiaohua Cheng, Shubo Chen, Yang Liu, Feng Yang, Jie Chen

**Affiliations:** ^1^ School of Tourism and Culinary Arts of Zhejiang Business College Hangzhou China; ^2^ School of Food Science and Biotechnology Zhejiang Gongshang University Hangzhou China

**Keywords:** antioxidant activity, enzymatic method, *in vitro* digestibility, peach gum polysaccharide, structural characterization

## Abstract

Research on peach gum polysaccharides (PGP) has recently surged; nevertheless, its utilization remains circumscribed by its elevated molecular weight. This investigation employed xylanase to depolymerize PGP, assessing the antioxidant potential and in vitro digestibility of the resultant emulsion. The analysis disclosed that the average molecular weight of the enzyme‐extracted PGP (EPGP) stood at 1.3 × 10^6^ Da. Characteristic absorption bands of EPGP were pinpointed through Fourier transform infrared spectroscopy. The monosaccharide composition of EPGP encompassed L‐rhamnose, D‐galactose, D‐mannose, D‐xylose, and L‐arabinose, with molar ratios of 0.4:0.7:6.6:30.8:61.5, correspondingly. Furthermore, EPGP exhibited superior antioxidant capabilities in contrast to water‐extracted PGP, elevating emulsification efficiency and free fatty acid liberation rates when juxtaposed with gelatin and Arabic gum. This exploration lays a foundational framework for the potential incorporation of PGP in food emulsions and pharmaceutical formulations.

## Graphical abstract

Xylanase was employed for the extraction of peach gum polysaccharide, resulting in the production of a low molecular weight polysaccharide with a molecular weight of 1.3 × 106 Da. Low molecular weight polysaccharides exhibit elevated antioxidant activity. EPGP demonstrates superior emulsification properties compared to AG and MG. EPGP showcases a heightened free fatty acid release rate.

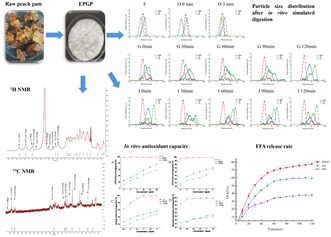

## 1 Introduction

Peach gum, a viscous exudate hydrocolloid secreted by *Prunus persica* (L.) Batsch (Rosaceae family), is predominantly harvested from cultivated orchards in major peach‐producing regions (Zeng et al. 2022). Peach gum polysaccharide (PGP) is a naturally occurring acidic heteropolysaccharide from the peach tree, primarily formed in response to injury (Li et al. 2018). Recent investigations have characterized DPG2, a novel arabinogalactan type II (AG II) polysaccharide derived from peach gum ( *Prunus persica* exudate), featuring a β‐(1 → 6)‐linked galactan backbone (Wei et al. 2023). Structural analysis revealed its heteropolysaccharide composition with molar ratios of mannose: glucuronic acid: galactose: xylose: arabinose = 4.64: 1.02: 2.61: 39.82: 3.89: 48.02. The macromolecule demonstrated a weight‐average molecular weight (Mw) of 5.21 × 10^5^ g/mol, exhibiting a flexible chain conformation with characteristic coil‐like tertiary structures. Subsequent studies identified three additional AG II variants (LP100R, LP10R, and LP5R) sharing the conserved β‐(1 → 6)‐galactan backbone architecture, distinguished by O‐3/O‐4 branched substitutions. These distinctive architectural features collectively contribute to the unique structure‐function relationships observed in peach gum polysaccharides (Zeng et al. 2022).

However, its high molecular weight and limited solubility at room temperature restrict its industrial use (Wu et al. 2020). Thus, reducing its molecular weight is crucial for enhancing its applicability. Depolymerization of polysaccharides can be achieved through chemical, enzymatic, or physical methods (Chen et al. 2024). Chemical methods, such as acid hydrolysis, often require harsh conditions, lack selectivity, and pose environmental risks. Physical methods need specialized equipment and generally yield low amounts of degraded product. In contrast, enzymatic hydrolysis offers advantages like cost‐effectiveness, mild conditions, high product activity, efficient extraction, energy conservation, and environmental friendliness (Chen et al. 2022, 2025).

Enzymatic hydrolysis not only lowers molecular weight but also modifies the structure by debranching lateral chains, thus broadening potential applications (Ma et al. 2018). Xylanases, a group of enzymes, are particularly effective in breaking down β‐1,4‐glycosidic bonds in the main chain through a synergistic action (Coutinho et al. 2020; Kaushal et al. 2021). Recent approaches to reduce PGP's molecular weight include water, alkali, and hydrogen peroxide extraction (Wei et al. 2019). However, enzymatic degradation remains underexplored. The polysaccharide fractions were isolated from enzymatically modified peach gum (EPGP) through an optimized xylanase‐assisted extraction protocol, followed by sequential purification employing DEAE‐52 cellulose anion‐exchange chromatography and Sephadex G‐200 gel filtration. Comprehensive structural analysis encompassed molecular weight determination via high‐performance gel permeation chromatography (HPGPC), functional group identification using Fourier‐transform infrared spectroscopic analysis, monosaccharide composition profiling through high‐performance liquid chromatography with pre‐column derivatization, conformational characterization by Congo red binding assay, and detailed molecular architecture elucidation employing one‐ and two‐dimensional nuclear magnetic resonance spectroscopy (^1^H, ^13^C, HSQC, HMBC).

This research aims to elucidate the structure–function relationship of EPGP to facilitate its application. Additionally, the behavior of EPGP‐based emulsions in various digestive environments (oral, gastric, and intestinal) was assessed using an in vitro simulated digestion model, providing insights into PGP's impact on fat digestion. This could pave the way for novel dietary and therapeutic applications.

## 2 Materials and Methods

### 2.1 Materials

Peach gum was sourced from Jinhua city of Zhejiang Province, China (119°38′41′′E, 29°05′32′′N) on 03/20/2023. The xylanase (Y35469, ≥ 2500 units/g), EC 3.2.1.8, CAS 37278‐89‐0, was purchased from Shanghai yuanye Bio‐Technology Co. Ltd. Chromatographic media including Sephadex G‐200 gel filtration matrix and DEAE‐52 anion‐exchange resin were sourced from Solarbio Science & Technology Co. Ltd. (Beijing, China). Monosaccharide reference compounds (D‐arabinose, D‐galactose, D‐glucose, D‐mannose, and D‐xylose) were acquired from Sigma‐Aldrich China (Shanghai Branch). All remaining chemical reagents and solvents employed in this study were certified analytical‐grade materials.

### 2.2 Enzyme‐Assisted Extraction and Column Chromatography Purification of PGP

The extraction protocol commenced with mechanical pulverization of raw peach gum exudate (40.1 g) to achieve 100‐mesh particle size. The powdered material underwent aqueous hydration (1:50, w/v) in 2 L deionized water under continuous agitation (24 h, 25°C). Enzymatic digestion was performed using xylanase (2% w/v loading) at optimal conditions (50°C, 4 h), followed by thermal enzyme inactivation (100°C, 10 min). The hydrolysate was subjected to sequential processing, centrifugation (3000 × g, 20 min, 4°C) for solid–liquid separation. The clarified liquid phase was retained and subjected to dual liquid–liquid extraction, followed by vacuum concentration (Rotavapor R‐300, Büchi). Protein contaminants were removed through chloroform‐butanol partitioning (Sevag protocol, 4:1 v/v), with subsequent oxidative decolorization using 3% H_2_O_2_. The polysaccharide was precipitated with alcohol, redissolved, and dialyzed in a 3 k Da bag at 4°C for 24 h, changing the water every 6 h. The purified biopolymer was ultimately converted to powder through freeze‐dried (−50°C), designated as enzyme‐processed peach gum polysaccharide (EPGP).

For primary fractionation, 100 mg EPGP was loaded onto DEAE‐52 cellulose anion‐exchange resin and eluted with stepwise NaCl gradients (0.1, 0.2, 0.3 mol/L) at a 1 mL/min flow rate. Active fractions detected by the phenol‐sulfuric acid assay were pooled and further resolved through Sephadex G‐200 gel permeation chromatography under isocratic elution (deionized water). Polysaccharide‐containing eluates (5 mL aliquots) were collected, concentrated, and cryodesiccated to obtain EPGP (Ye et al. 2021).

### 2.3 HPLC Analysis of PGP Molecular Weight Distribution

PGP molecular weight distribution was measured by HPLC with a differential refractive detector (Agilent 1100, Shanghai, China). The experimental conditions were established as described below: The mobile phase was composed of 50 mmol/L Na₂SO₄, which was filtered through a 0.45 μm membrane. A refractive index detector was employed, and the column temperature was maintained at a constant 30°C. The injection volume was set at 50 μL. The mobile phase flowed at a rate of 0.5 mL/min through a Waters Ultrahydrogel Linear column (10 μm, 7.8 × 300 mm). Standard extran solutions with different molecular weights (5000; 25,000; 150,000; 670,000 Da) were prepared at a concentration of 5 mg/mL using a flow‐matching technique. These solutions were then mixed in appropriate proportions. The polysaccharide sample, having a concentration of 5 mg/mL, was filtered through a 0.22 μm membrane prior to HPLC analysis. The molecular weight distribution of the crude polysaccharides was determined by the peak area uniform method.

### 2.4 Analysis of Monosaccharide Composition

A total of 10 mg of EPGP was accurately weighed and transferred into an Eppendorf tube, followed by the addition of 2 mL of 2 mol/L TFA. The mixture was sealed under a nitrogen atmosphere and hydrolyzed at 90°C for 8 h. After hydrolysis, the sample was allowed to cool, and adding 2 mL of methanol, excess TFA was eliminated through repeated nitrogen drying. Standards for monosaccharides (arabinose, D‐mannose, D‐galactose, rhamnose, D‐glucose, D‐xylose, and galacturonic acid) were prepared at a concentration of 1 mg/mL. Each standard 200 μL solution was mixed with 0.3 mol/L NaOH and 0.5 mol/L PMP, and 100 μL, respectively, in methanol, heated at 70°C for 30 min, cooled, and then neutralized with 100 μL of 0.3 mol/L HCl. The mixtures were extracted with 1 mL of trichloromethane, vortexed, and centrifuged. After discarding the organic layer, the extraction was performed three additional times. The aqueous phase was then passed through a 0.22 μm filter.

Chromatographic analysis utilized a mobile phase made up of phosphate buffer (pH 6.8) and acetonitrile in an 85:15 proportion, with a 1.0 mL/min flow rate and a 20 μL injection volume. Detection was set at 250 nm to achieve optimal monosaccharide separation.

### 2.5 FT‐IR, Congo Red Test and NMR Analysis

A 2 mg portion of dried EPGP was combined with 200 mg of KBr, formed into a pellet, and examined using FTIR spectroscopy across a spectrum of 4000–400 cm^−1^ with a resolution set at 4 cm^−1^. The conformational structure of EPGP was assessed by the Congo Red test as Niu et al. (2021). Here, 2 mL of 80 μmol/L Congo Red and varying concentrations of NaOH (0.0–0.5 mol/L) were added to 2 mL of a 2.5 mg/mL polysaccharide solution. The maximum absorbance wavelength (λ_max_) was measured between 400 and 600 nm and plotted against NaOH concentration. For NMR analysis, 30 mg of EPGP was lyophilized with D_2_O, dissolved in 0.6 mL D_2_O, and passed through a 0.22 μm filter membrane. Spectra for ^1^H NMR, ^13^C NMR, COSY, HMQC, and HMBC spectra were obtained using a Bruker Advance M NMR spectrometer at 298 K, with data processed by Bruker TopSpin software.

### 2.6 Radical Scavenging Assays for Evaluating Antioxidant Activities

Based on Tian et al. (2020), this method involves reacting polysaccharide solutions (3–18 mg/mL) with 0.1 mM DPPH in ethanol for 30 min in the dark at room temperature, with absorbance measured at 517 nm. Modified from Mutaillifu et al. (2020), this procedure entails preparing an ABTS radical solution, which is then reacted with polysaccharide samples (1–6 mg/mL) for 30 min at 25°C, and absorbance measured at 734 nm. Adapted from Wang et al. (2022), this assay mixes polysaccharide solutions (0.5–3 mg/mL) with FeSO_4_, salicylic acid in ethanol, and H_2_O_2_, held at 37°C for 30 min, with absorbance recorded at 510 nm. Following Su and Li (2020), this method involves warming a Tris–HCl buffer mix, adding varying concentrations of polysaccharides and pyrogallol, reacting for 6 min, halted by HCl, with absorbance measured at 325 nm.

### 2.7 Analysis of Emulsion Digestion in Simulated Oral, Gastric, and Intestinal Fluids

For the EPGP emulsion, concentrations were set at 4% (w/v) for the continuous phase and 4% (v/v) for the oil phase, with 4 min of ultrasonic treatment at 360 W. The arabic gum (AG) emulsion had a continuous phase of 8% (w/v) and an oil phase of 4% (v/v), ultrasonicated for 4 min at 600 W. The gelatin gum (MG) emulsion featured a 2% (w/v) continuous phase and a 2% (v/v) oil phase, also ultrasonicated for 4 min at 600 W. Initially, to coarsely prepare the emulsions, the continuous phase was combined with soybean oil using a homogenizer (Ultra‐Turrax T10, IKA, Germany) at 10,000 rpm for 2 min, followed by ultrasonic treatment to finalize the emulsions.

Simulated oral digestive fluid (SSF) was prepared as specified in Table 1. Fresh emulsion was allocated into 10 mL centrifuge tubes (1 mL per tube), mixed with an equal volume of SSF, and pH was set to 6.8 using 1 mol/L NaOH. After taking one sample for immediate analysis, the rest were incubated at 37°C (100 rpm) and sampled after 3 min. Simulated gastric fluid (SGF) was set to pH 2 with 1 mol/L HCl and enriched with pepsin (3.2 g/L). This was then mixed with the orally digested sample, pH re‐adjusted to 2, and incubated at 37°C (100 rpm) for 120 min. Sampling occurred at 0, 30, 60, 90, and 120 min. Simulated intestinal fluid (SIF), containing trypsin (3.2 g/L) and bile salt (2.5 g/L), was prepared following Table 1 guidelines. In post‐gastric digestion, prior to adding SIF, pH was quickly corrected to 7.0 with 1 mol/L NaOH. The mixture was subsequently incubated at 37°C with a shaking speed of 100 rpm for 120 min, with sampling at the same intervals as gastric digestion (Table 2).

**TABLE 1:** Preparation table of digestive liquid.

Reagent	Concentration (g/L)		
	SSF	SGF	SIF
KCl	37.3	17.25	17
KH_2_PO_4_	9.25	2.25	2
Bis‐Tris	17	32.5	106.2
MgCl_2_	1.25	1	2.75
CaCl_2_	0.063	0.0125	0.1
NaCl	—	29.5	24

Abbreviations: SGF, simulated gastric fluid; SIF, simulated intestinal fluid; SSF, simulated oral digestive fluid.

**TABLE 2:** Assignments of ^1^H and ^13^C for EPGP.

Sugar residue		Chemicla shift (δ, ppm)					
		H1/C1	H2/C2	H3/C3	H4/C4	H5/C5	H6/C6
A	1,3,6‐β‐D‐Galp	4.46/103.16	3.98/76.50	3.82/81.78	3.64/82.37	3.78/73.30	4.05/68.28
B	1,3‐α‐L‐Araf	5.09/104.80	3.54/71.80	4.66/84.86	3.91/75.82	3.77/74.90	4.01/69.90
C	1,6‐β‐D‐Galp	4.57/103.20	3.56/69.74	3.67/73.00	4.09/68.22	3.64/70.32	3.75/67.76
D	1,3‐β‐D‐Galp	4.37/103.11	3.79/76.50	3.89/83.60	3.97/69.90	3.64/69.30	3.82/68.07
E	α‐L‐Rhap	5.07/107.50	4.99/100.94	3.87/83.50	4.01/76.70	3.78/73.70	1.15/16.60
F	1,2,3‐α‐D‐Galp	5.01/101.44	4.33/69.60	3.82/67.30	4.34/81.20	3.83/72.50	3.41/75.60
G	1,3,4‐α‐D‐Galp	4.92/99.79	3.72/69.70	3.92/70.80	4.14/78.10	3.89/73.80	3.77/62.30
H	β‐D‐Manp	4.65/100.87	4.02/71.90	3.73/72.40	3.96/71.10	3.33/73.60	3.47/62.30
I	1,α‐L‐Araf	5.43/108.05	5.25/109.86	5.09/107.09	5.06/106.66	5.00/107.08	3.92/63.40
J	1,4)‐β‐D‐Xyl	4.42/101.96	3.34/69.44	3.60/72.81	3.69/74.91	3.92/63.40	3.41/70.50

The pH during intestinal digestion was maintained around 7 by adding 0.1 mol/L NaOH via the pH‐stat method. The volume of NaOH used was recorded to calculate the release of free fatty acids (FFAs) using the formula:

$$\text{FFAs}\;\left(\%\right)=\frac{V\left(\text{NaOH}\right)\times C\left(\text{NaOH}\right)\times M\left(\text{oil}\right)}{2\times m\left(\text{oil}\right)}\times 100\%$$

### 2.8 Particle Size Analysis

A defined quantity of the emulsion was placed into the sample chamber of the Mastersizer (2000, Malvern, UK). The emulsion was dispersed uniformly at 2000 rpm within the measurement chamber. Utilizing a dual‐wavelength detection system with blue and red light, the setup enhanced both sensitivity and accuracy. The Mastersizer software was used to analyze particle size and distribution, measuring the volume mean diameter D(4,3) in micrometers (μm).

### 2.9 Zeta Potential Measurement

To minimize multiple scattering effects, the emulsion sample was diluted 100 times with deionized water. Zeta potential was measured using a Zetasizer Nano (ZS 90, Malvern, UK) at 25°C. Measurements were taken three times using the DTS‐7010 capillary colorimeter, and the average value was calculated for further correlation analysis.

### 2.10 Optical Microscope Observation

The microstructure of emulsion was examined with an optical microscope (DM3000, Leica Microsystems GMBH). A 20 μL emulsion was placed on a glass slide, covered with a coverslip, and observed.

### 2.11 Statistical Analyses

All experiments were performed three times. Statistical analysis was conducted using SPSS software (Version 27.0, IBM Corp.), with results expressed as mean ± standard deviation. Graphical representations were created using Origin 2021 (OriginLab Corp.) following academic illustration standards.

## 3 Results and Discussion

### 3.1 Characterization and Molecular Weight Analysis of EPGP

**FIGURE 1:**
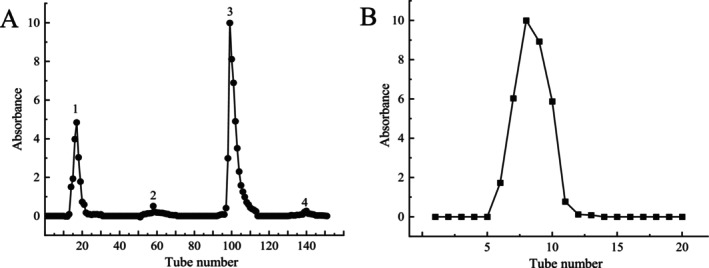
Elution profiles of EPGP from anion‐exchange chromatography (A) and size‐exclusion chromatographic separation (B).

**FIGURE 2:**
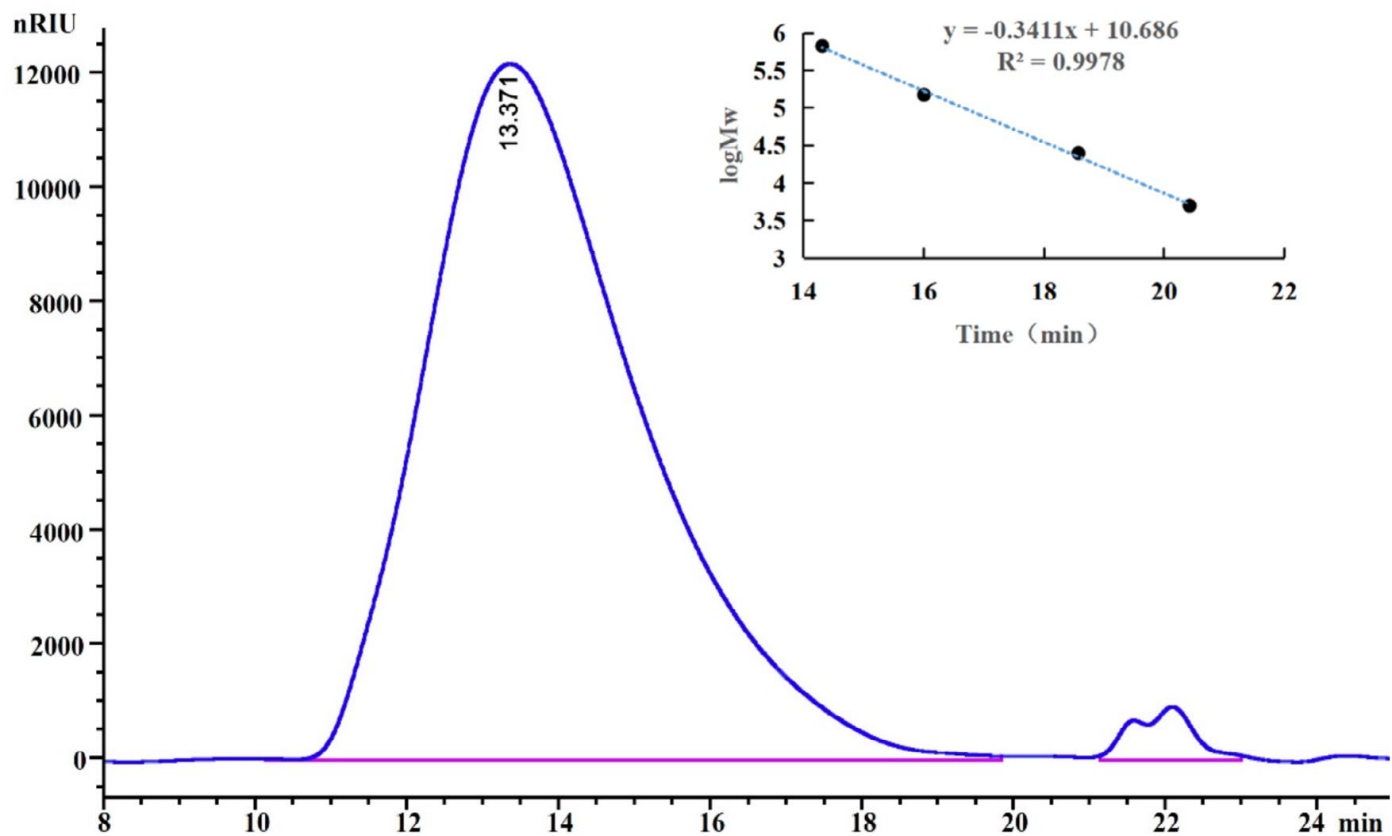
HPLC analysis of purified EPGP under optimized conditions.

After extraction, the crude polysaccharide underwent ethanol precipitation, Sevage deproteinization, and lyophilization. It was then separated using DEAE‐52 anion‐exchange chromatography and further purified with a Sephadex G‐200 gel filtration column (Figure 1). The molecular weight of EPGP, determined by HPLC, showed a homogeneous peak and was calculated to be 1.3 × 10^6^ Da (Figure 2), significantly lower than previously reported values (Simas et al. 2008; Wei et al. 2019), suggesting that xylanase extraction effectively reduced the molecular weight.

**FIGURE 3:**
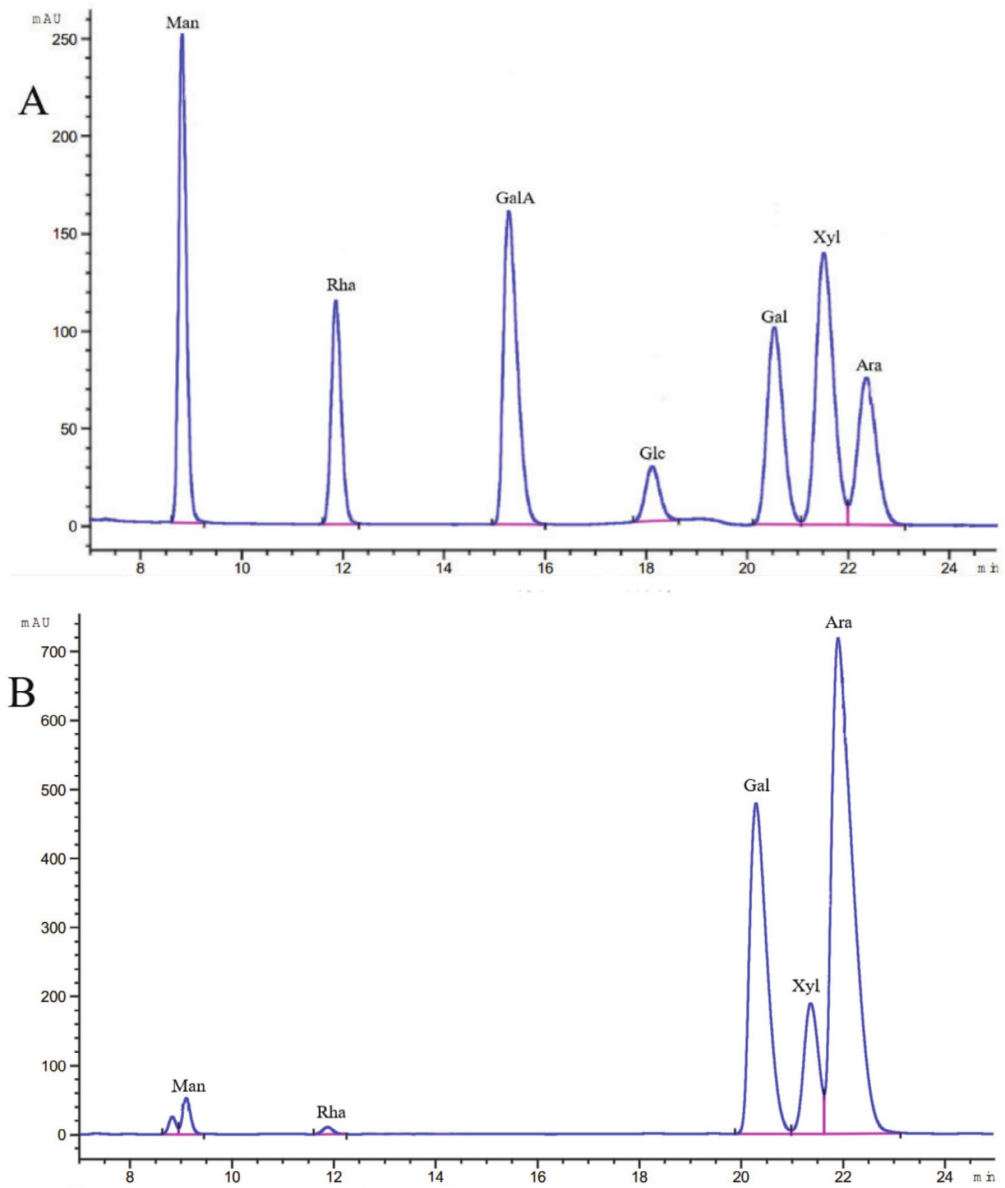
Monosaccharide composition analysis by HPLC with pre‐column PMP derivatization: (A) Standard monosaccharides; (B) Hydrolysate of EPGP.

The monosaccharide components of EPGP were determined by HPLC, as presented in Figure 3, included mannose, rhamnose, galactose, xylose, and arabinose in a molar ratio of 0.7:0.4:30.8:6.6:61.5. EPGP was characterized as an acid hetero‐polysaccharide predominantly composed of galactose, xylose, and arabinose. A previous study (Simas‐Tosin et al. 2010) reported that a PGP consisted of three monosaccharides: arabinose, xylose, and mannose. Despite the similarity in monosaccharide compositions, the monosaccharides with the highest levels were distinct. Furthermore, the antioxidant activity of polysaccharides is influenced by their monosaccharide composition (Sasikumar et al. 2023). The antioxidant effects of polysaccharides were tied to their chemical composition, the types of monosaccharides present, and their molecular weight (Janardhanan et al. 2024).

### 3.2 FT‐IR Analysis and Congo Red Test

**FIGURE 4:**
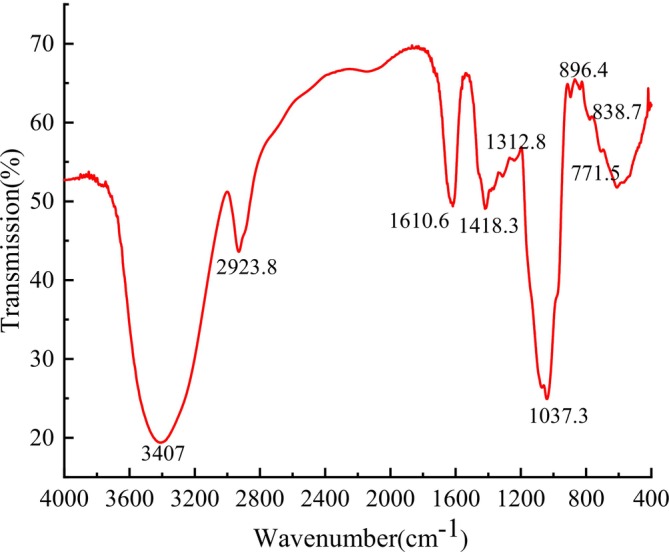
FT‐IR spectroscopic characterization of EPGP.

FT‐IR spectroscopy was employed to determine functional groups in EPGP using the KBr pellet method, as illustrated in Figure 4. The spectrum displayed characteristic polysaccharide absorption peaks between 4000 and 500 cm^−1^. A general absorption band at 3407 cm^−1^ corresponds to the stretching vibration of OH groups (Lajili et al. 2019), while the absorption peak at 2923 cm^−1^ indicates a typical C‐H stretching vibration (Dammak et al. 2019). The absorption peak, approximately at 1418 cm^−1^, is associated with the symmetric stretching of the carboxylate anion (Zhao et al. 2020). The signal near 1037 cm^−1^ relates to the distinctive peak of pyranose, particularly COC and COH stretching bands. Absorption peaks at approximately 896 and 838 cm^−1^ correspond to β‐ and α‐end isomers of pyranose rings (Zhang et al. 2021), respectively, indicating the presence of α‐glucoside and β‐glucoside bonds. Additionally, the absorption peak at 771 cm^−1^ confirms the presence of pyran structure, as the weak absorption is due to vibration involving symmetric stretching in α‐pyran. Consequently, FT‐IR spectrum analysis revealed a typical absorption peak for the purified EPGP.

**FIGURE 5:**
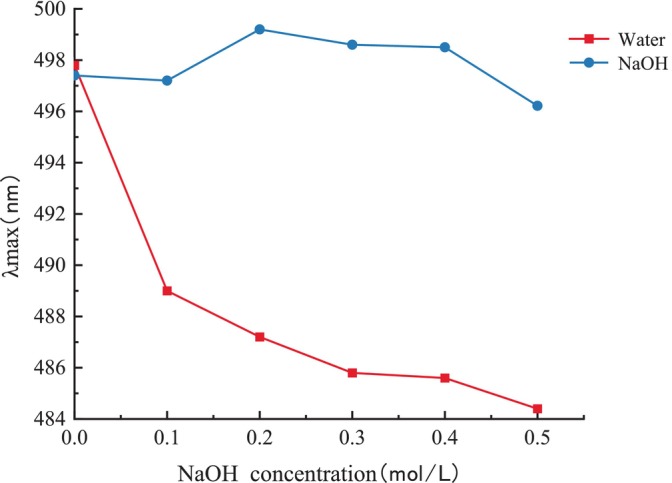
UV–Vis spectrophotometric determination of maximum absorption wavelength (λ_max_) for EPGP in alkaline solutions.

Congo Red is capable of forming complexes with polysaccharides that possess a triple‐helical structure. In contrast to Congo Red by itself, the λ_max_ of the complex undergoes redshift and exhibits a metastable state within a specific range of NaOH concentration (Figure 5), reflecting a characteristic change in the absorption wavelength (Guo et al. 2021). The interaction between polysaccharide and Congo Red demonstrated characteristic changes in λ_max_ within the range of 0–0.5 mol/L NaOH, indicating that PGP possesses a relatively stable triple‐helix structure (Mei et al. 2020).

### 3.3 NMR Spectroscopy Analysis

Residues with anomeric protons chemical shifts exceeding δ_H_ 4.95 ppm are generally in the α‐configuration, while chemical shifts below δ_H_ 4.95 ppm indicate the presence of β‐configuration (Yao et al. 2021). In the ^13^C NMR spectrum, resonances within the region of δ_C_ 95.0–110.0 ppm are attributed to the anomeric carbon atoms, with the typical chemical shift range for the α‐configuration being δ_C_ 90–102 ppm (Liu et al. 2021). ^1^H NMR spectra revealed that PGP was composed of α‐ and β‐glycosides; however, only one ectopic hydrogen proton chemical shift exceeded δ 5.0 mg/L, specifically at δ 5.09 mg/L, indicating that PGP predominantly consisted of β‐type glycosides. Additionally, two weak peaks in the region of 98–103 ppm were observed, corresponding to the results of ^1^H NMR analysis. In the ^1^H spectrum, the signal for α‐Glc‐p appeared at δ_H1_ 5.35 ppm (Dujnič et al. 2021). The residual hydrogen peak of the solvent, D_2_O, was observed at δ_H1_ 4.70 ppm. The ectopic hydrogen proton signal appeared between δ 4.9 and 5.2 ppm, suggesting that the sample was pyranose. Additional evidence for the pyranose form is the absence of a signal peak at δ 83–88 ppm in the ^13^C NMR spectrum, which indicates the absence of furanose. A signal peak below δ 80 ppm supports that the sample is pyranose type (Liu et al. 2021). The resonance at δ 4.38/103.2 ppm corresponds to the anomeric proton and anomeric carbon of β‐1,4‐Galp and β‐T‐Galp, respectively. Signals at δ 5.06/106.66 and 5.00/107.08 ppm were assigned to α‐T‐Araf and α‐1,5‐Araf. The range of δ 175–170 ppm in the ^13^C spectrum was assigned to the carboxyl carbon of Gal‐p‐A, indicating the presence of uronic acid and methyl uronic acid (Wan et al. 2021). In the ^1^H NMR spectrum, between 1.1 and 1.3 ppm was the chemical shift for hydrogen on the carbon of the methyl signal, while in the ^13^C NMR, the carbon signal for the methyl group was observed between 16 and 18 ppm, indicating the presence of methyl groups on the terminal carbon. The signal peak between 3.3 and 3.5 ppm in the ^1^H NMR spectrum was attributed to the presence of oxygen‐methyl groups, which was further confirmed by the signal at 61 ppm in the ^13^C NMR spectrum (Yang et al. 2021).

**FIGURE 6:**
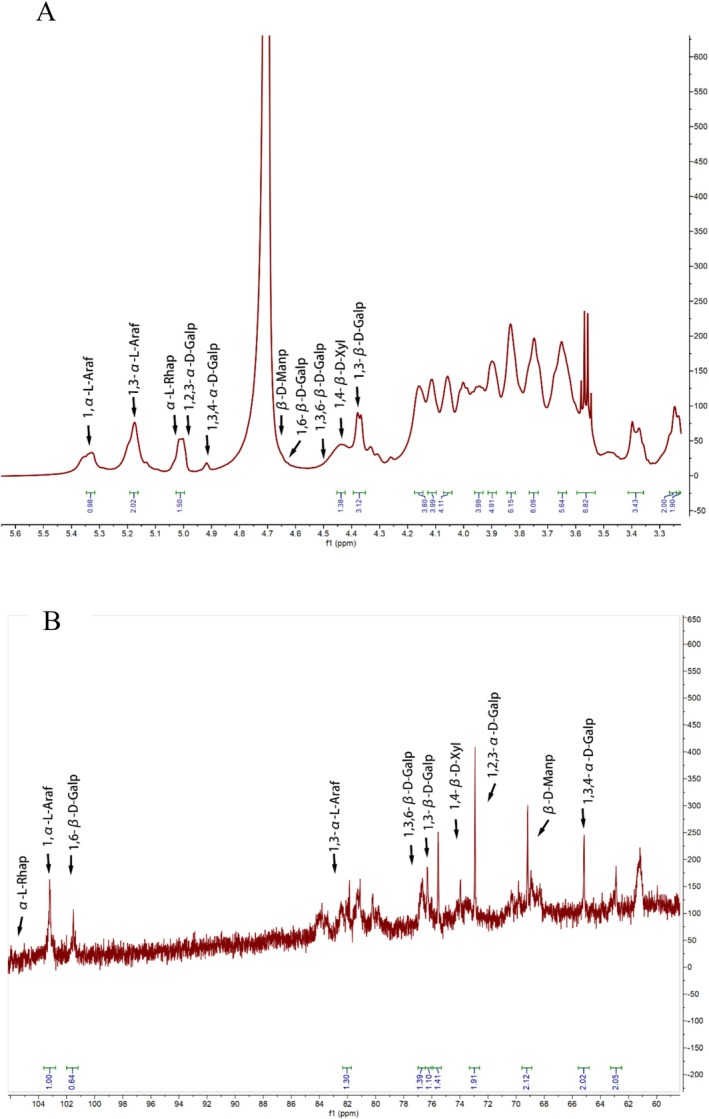
^1^D NMR spectroscopic analysis of EPGP: (A) ^1^H NMR spectrum and (B) ^13^C NMR spectrum.

The ^13^C NMR spectrum (Figure 6) was unable to determine the number of sugar residues because of overlapping signals and the low presence of certain monosaccharide residues in the polysaccharide samples. Consequently, identifying the type of primary glycosidic bond and the exact positions of the corresponding H and C atoms with the help of 2D NMR correlation spectra was required.

**FIGURE 7:**
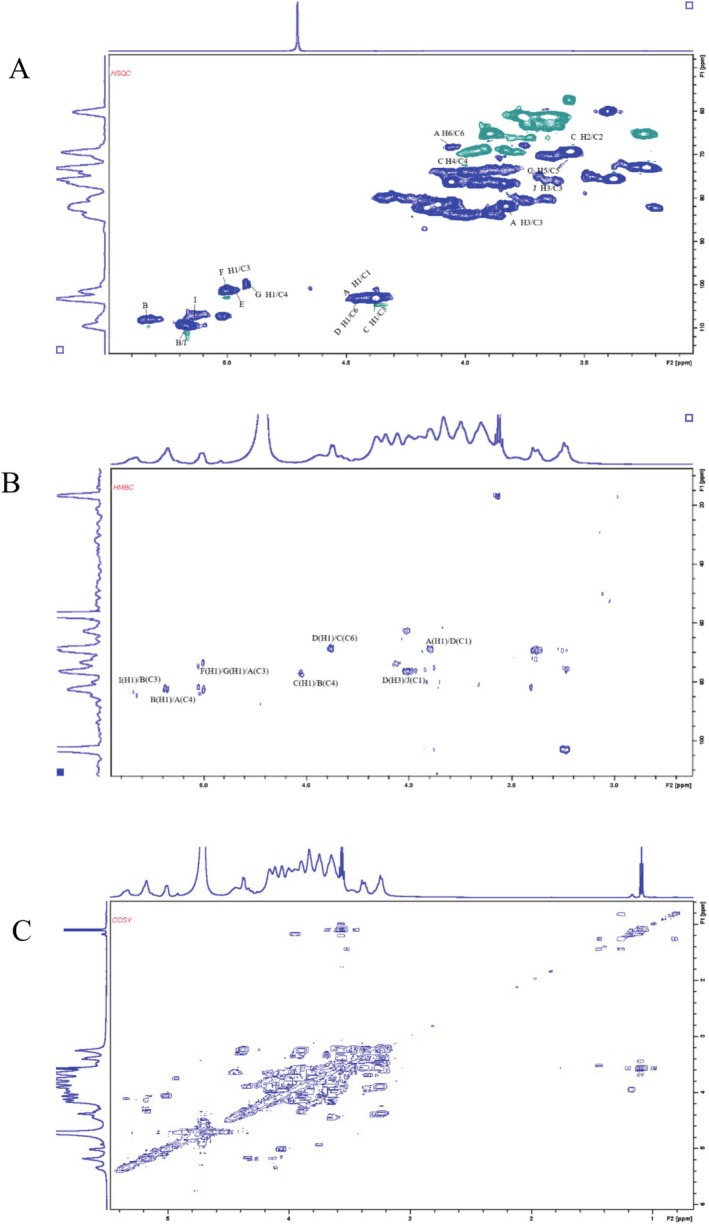
Two‐dimensional (2D) homonuclear and heteronuclear correlation spectroscopy of EPGP: (A) ^1^H‐^13^C HSQC; (B) ^1^H‐^13^C HMBC; (C) ^1^H‐^1^H COSY.

The ^1^H NMR spectrum (Figure 6) at 4.43 ppm was attributed to β‐D‐Galp, with the corresponding residual carbon appearing at 103.05 ppm in the heteronuclear singular quantum correlation (HSQC) spectrum. By combining data from COSY and HSQC spectra, the chemical shifts for H‐1/C‐1 (δ 4.46/103.16 ppm), H‐3/C‐3 (δ 3.82/81.78 ppm), and H‐6/C‐6 (δ 4.05/68.28 ppm) of sugar residue A were observed to shift to lower fields, indicating that substitution occurred. Furthermore, cross‐peaks between H‐1 (4.38 ppm) and C‐6 (68.44 ppm) were identified in the HMBC spectrum（Figure 7). These data suggest that the linkage mode of sugar residue (A is →3,6)‐β‐D‐Galp‐(1→, and the main chain structure is constructed as →6)‐β‐D‐Galp‐(1→ (Alba et al. 2020). The cross peak between H‐1 (5.33 ppm) and C‐3 (84.86 ppm) in HMBC spectrum indicated the presence of →3‐α‐L‐Araf‐(1→ linkage mode (residues B). Combining data from HSQC and COSY, 3.56/69.74, 4.09/68.22, 3.64/70.32, and 3.75/67.76 ppm, corresponding to H‐2/C‐2, H‐4/C‐4, H‐5/C‐5, and H‐6/C‐6, respectively, demonstrated the presence of →6)‐β‐D‐Galp‐(1→ presence (residues C). Besides, residual signal at 4.36/103.11 ppm was attributed to →3)‐β‐D‐Galp‐(1→ (residues D). Similarly, other structural residues were identified as follows: Residue E: α‐L‐Rhap (4.99/100.94), Residue F: →2,3)‐α‐D‐Galp‐(1→ (5.01/101.44 ppm), Residue G: →3,4)‐α‐D‐Galp‐(1→ (4.92/99.79) (Košálová and Hromádková 2019), Residue H: β‐D‐Manp (4.65/100.87), Residue I: α‐L‐Araf‐(1→ (5.09/107.09 ppm), and Residue J: →4)‐β‐D‐Xyl‐(1→ (4.42/101.96)) (Sigida et al. 2020).

In the HMBC spectrum（Figure 7), the H‐1 (5.36 ppm) of residue I and C‐3 (83.87 ppm) of residue B, as well as H‐1 (5.19 ppm) of residue B and C‐4 (82.37 ppm) of residue A, exhibited strong cross peaks. These findings suggest that the branched‐chain α‐L‐Araf‐(1,3)‐α‐L‐Araf‐(1→ is connected to the O‐3 position of the main chain. Additionally, the H‐1 (5.33 ppm) of sugar residue I was correlated with C‐3 (85.07 ppm) of sugar residue B, suggesting that the α‐L‐Araf‐(1 → 3)‐β‐D‐Man branched chain is connected to the O‐3 position of the main chain. Furthermore, the H‐1 of residue E and H‐1 of residue H were correlated with C‐6 of residue C in the HMBC spectrum, indicating that α‐L‐rhap‐(1→ and β‐D‐Manp‐(1→ are connected to →6)‐β‐D‐Galp‐(1→（Table 2)).

In the HMBC spectrum（Figure 7), H‐1 (4.37 ppm) of residue D was correlated with C‐6 (68.07 ppm) of residue C, and H‐1 (4.57 ppm) of residue C was correlated with C‐4 (75.82 ppm) of residue B. These data suggest that →6)‐β‐D‐Galp‐(1→ and →3)‐β‐D‐Galp‐(1→ are attached to →3)‐α‐L‐Araf‐(1→. Additionally, the H‐1 (5.01 ppm) of residues F/G was correlated with C‐3 (74.16 ppm) of sugar residue A in the HMBC spectrum, demonstrating that sugar residues E/F were linked to O‐4 of residues A. In summary, PGP is an arabinogalactan with β‐(1 → 6)‐galactose as the main chain.

### 3.4 Antioxidant Activities

#### 3.4.1 DPPH Radical Scavenging Activity

**FIGURE 8:**
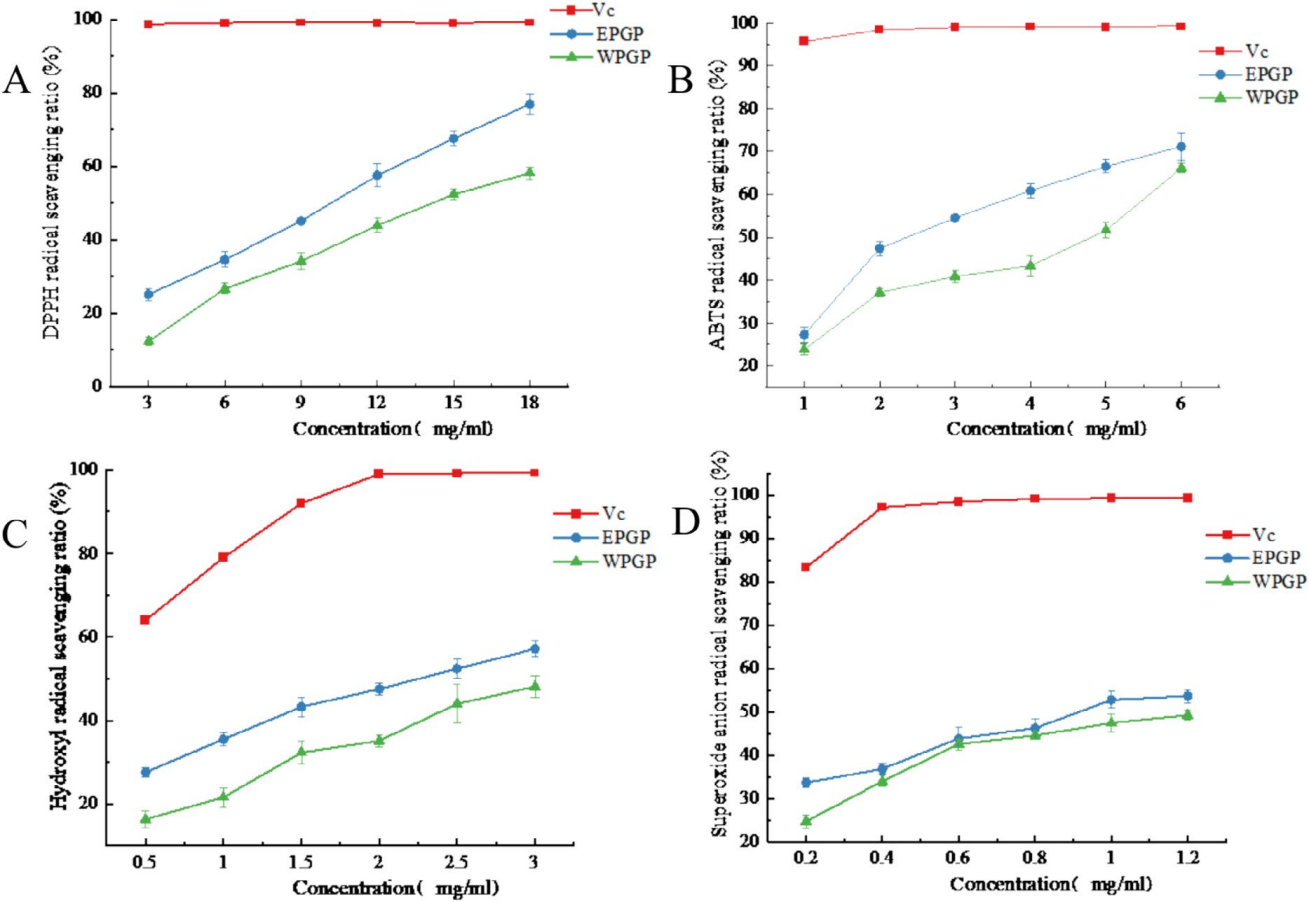
In vitro antioxidant capacity evaluation of EPGP: (A) DPPH; (B) OH•; (C) ABTS; (D) O_2_^−^•.

The efficacy of EPGP and WPGP in scavenging DPPH free radicals was elucidated in Figure 8A. When the polysaccharide concentration rose from 3 to 18 mg/mL, the scavenging efficacy of EPGP and WPGP surged from 25.13% and 12.32% to 76.94% and 58.2%, sequentially. These outcomes signified an amplified scavenging efficiency towards DPPH free radicals with the mounting polysaccharide concentration. Moreover, EPGP showcased heightened DPPH radical scavenging prowess when juxtaposed with WPGP.

#### 3.4.2 OH Radical Scavenging Activity

Figure 8B illustrated the OH radical scavenging capacities of EPGP and WPGP. Within the 0.5–3 mg/mL range, both EPGP and WPGP displayed progressively heightened scavenging efficiency towards OH radicals with increasing polysaccharide concentrations. Nevertheless, their efficacy in OH radical scavenging fell short in comparison to VC. Notably, EPGP, characterized by a lower molecular weight, showcased augmented scavenging prowess towards OH radicals. This observation indicated that the decrease in polysaccharide molecular weight aids in more effectively removing OH radicals. When the concentration is 0.5 mg/mL, EPGP and WPGP exhibited OH radical scavenging capacities of 27.6% and 16.31%, respectively. Upon escalation to 3 mg/mL, EPGP and WPGP demonstrated scavenging rates of 57.13% and 48.1%, correspondingly. The comparison underlined EPGP's approximately 10% superiority in OH radical scavenging compared to WPGP. The observed effect may stem from the improved water solubility and increased surface area of the lower molecular weight samples obtained using enzyme‐assisted methods, thus augmenting their ability to interact with free radicals.

#### 3.4.3 ABTS Radical Cation Scavenging Activity

The scavenging activity of polysaccharides against the ABTS radical cation is demonstrated in Figure 8C. EPGP and WPGP displayed remarkable prowess in combating the ABTS cationic radical, with their scavenging capabilities aligned with concentration gradients. Specifically, at a 6 mg/mL polysaccharide concentration, EPGP and WPGP showcased an ABTS cationic radical scavenging potency of 71.19% and 66.18%, respectively. Notably, at polysaccharide concentrations of 1 and 6 mg/mL, both EPGP and WPGP demonstrated comparable abilities in scavenging ABTS cationic radicals; yet consistently, EPGP exhibited superior scavenging efficacy compared to WPGP. To determine the antioxidant activity of polysaccharides, the ABTS free radical scavenging abilities have been thoroughly investigated. The scavenging activity exhibited by native polysaccharides and their acetylated derivatives demonstrates significant dose‐dependent characteristics, with efficacy positively correlated with compound concentration (Thimmaraju et al. 2023).

#### 3.4.4 O^2^ ^−^ Radical Scavenging Activity Assay

The capacity of EPGP and WPGP to scavenge O^2−^ was illustrated in Figure 8D. The findings revealed that both EPGP and WPGP possess the ability to scavenge O^2−^. Notably, as the concentration of polysaccharides increased, their effectiveness in combating O^2−^ saw a significant boost. Figure 8D highlighted that the O^2−^ radical scavenging potential of EPGP and WPGP falls notably short in comparison to VC. While the O^2−^ radical scavenging capabilities between EPGP and WPGP appear similar, WPGP consistently registered a lower capacity when compared to EPGP. With a polysaccharide concentration of 1.2 mg/mL, EPGP and WPGP showed the O^2−^ radical scavenging efficiencies of 53.67% and 49.24%, respectively, with only a marginal difference of 4.43% separating EPGP and WPGP.

### 3.5 Characterization of Emulsion and Digestion Stage

#### 3.5.1 Particle Size Analysis

**FIGURE 9:**
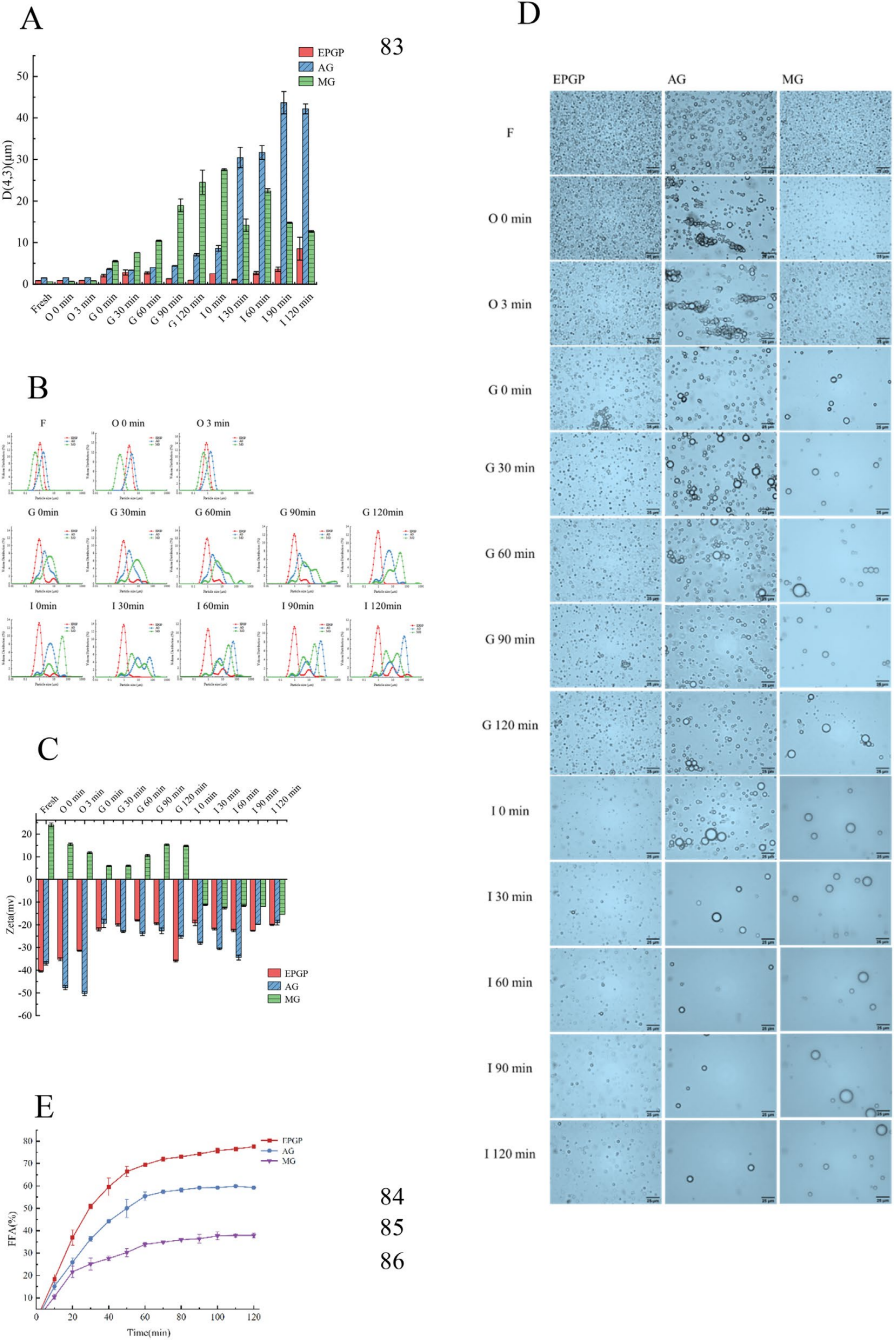
Physicochemical characterization of EPGP emulsion and digestion stage. (A) Particle size; (B) Particle size distribution; (C) Zeta potential; (D) Microstructure; (E) FFA release rate.

The initial stage analysis (Figure 9A) indicated no significant differences in particle sizes among EPGP (0.86 μm), AG (1.51 μm), and MG (0.57 μm) emulsions. Post oral digestion, a slight rise in particle size was observed in the three emulsions, with minimal variance. The particle size distribution, as depicted in Figure 9B, exhibited a distinct inverted “V” shape with a singular peak, suggesting uniform particle distribution and effective emulsification. During gastric digestion, the mean particle size within the emulsions further increased, with the successive average sizes for EPGP, AG, and MG emulsions recorded as 2.06, 3.66, and 1.37 μm, respectively. Notably, EPGP experienced a shift in its distribution peak to the right, possibly due to acidic gastric fluid‐induced emulsion aggregation. Meanwhile, the particle size of MG emulsion continued to grow, potentially attributed to pepsin's enzymatic hydrolysis. Coalescence of oil droplets during gastric digestion led to larger particle formation, elevating the mean particle size of the emulsions. Subsequent to gastric digestion, a significant rise in particle size and broadening of particle size distribution were observed, with AG and MG displaying distinctive dual peaks.

Upon closer inspection, MG's protein‐rich composition facilitated pepsin‐induced protein molecule breakdown, fostering aggregation between emulsion droplets. This resulted in a conspicuous prominence of larger particles within the particle size distribution. EPGP, distinguished by stable particle size distribution, likely owed its resilience to gastric fluid digestion to increased electrostatic repulsion between emulsion particles. Further into the study, following 120 min of intestinal fluid digestion, the particle size of the EPGP emulsion surged to 8.53 μm. Contrastingly, the AG emulsion witnessed a steady rise in particle size over digestion time, peaking at 43.67 μm. Intestinal digestion engendered a marked increase in emulsion droplet size, with EPGP presenting a distribution peak at 10 μm post‐digestion, potentially attributed to ion presence in intestinal fluid inducing emulsion instability. The AG emulsion exhibited an altered particle size distribution post‐digestion, with indications of droplet aggregation influenced by porcine bile salts and trypsin binding, leading to possible emulsion destabilization. The particle size distribution analysis of the MG histone emulsion postintestinal digestion positioned it slightly to the left in comparison to the AG group, indicative of a smaller particle size post gastric fluid digestion.

#### 3.5.2 Zeta Potential Analysis

The surface charge variations of oil droplets, affected by the type of emulsifier and stages of in vitro digestion, were assessed using Zeta potential measurement. Initial potential assessments (Figure 9C) revealed that EPGP and AG emulsions exhibited negative values below −30 mV, while MG displayed positive potentials. This divergence is attributable to the negatively charged nature of PGP and acacia gum as polysaccharides, contrasted by the positive charge of gelatin, a polysaccharide component. Furthermore, the predominant colloid within each emulsion system notably dictated its potential profile.

Post simulated oral digestion, a rise in the absolute potential of EPGP and AG emulsions likely resulted from the presence of diverse salt ions in oral fluid, amplifying the system's negative charge. Conversely, the positively charged MG emulsion saw an increase in absolute potential alongside an overall reduction in emulsion potential. Subsequent infusion of gastric fluid induced significant modifications in the charge characteristics of each emulsion system. The potential readings for EPGP, AG, and MG systems were −22, −19.33, and 5.93 mV, respectively. These readings notably declined from the previous stage, primarily owing to varied gastric environmental factors like ionic strength, pH levels, and enzyme activity. The gastric fluid's acidity (pH 2) diminished the droplet surface's negative charge, consequently plunging the emulsion droplets' absolute potential. This attenuation led to weakened electrostatic repulsion amid emulsion droplets, fostering particle aggregation, flocculation, and an upsurge in particle size. Concurrently, heightened ionic strength shielded a portion of the surface charge, diminishing electrostatic interactions within the emulsion system.

Upon introduction of intestinal digestive fluid, the potential of AG and MG emulsions transitioned from −25.30 and 14.80 to −28.10 and − 11.20 mV, respectively. This alteration denotes an escalation in negative charge within each digestive milieu, notably evident in MG emulsion's shift from positive to negative potentials. This inversion is ascribed to the prevalent negatively charged molecules in intestinal fluid, such as bile salts, phospholipids, and others. Additionally, the release of negatively charged FFA during fat digestion promotes the formation of negatively charged polymer groups facilitated by phospholipids and bile salts.

#### 3.5.3 Optical Microscope Observation Analysis

The microstructure of the emulsion was scrutinized through a light microscope to delve into structural changes during digestion. As illustrated in Figure 9D, initial emulsions manifested a microstructure resembling spherical droplets, with dispersed droplets across all samples suggesting an absence of flocculation or coalescence, alongside small particle sizes. Notably, the AG emulsion, stabilized by its emulsifier, exhibited larger particle diameters in alignment with particle size analysis outcomes. Following the addition of the oral digestive solution, signs of aggregation surfaced in the AG emulsion, whereas other emulsions experienced dilution without notable alterations. This aligns with Silletti et al. (2008) findings, indicating that emulsions boasting a strong negative charge resist flocculation in the presence of saliva, whereas those with a weaker negative charge may undergo reversible flocculation.

Amid gastric digestion, minor emulsion aggregation in EPGP was observed, likely influenced by salt ions in the digestive milieu. Nonetheless, the emulsion particle structure remained largely undamaged, sustaining integrity and exhibiting commendable stability throughout gastric digestion. Serving as a shield for stomach pH‐sensitive fat‐soluble bioactive compounds like retinol, these particles preserve their structural integrity. In protein‐rich MG, pepsin action led to significant emulsion particle digestion, leaving behind scant intact particles visible through the optical microscope. Post‐enteric digestion, visible emulsion particles diminished further due to intestinal fluid dilution. EPGP emulsion droplet sizes held steady with no notable changes or aggregation observed. Conversely, numerous emulsion particles persisted in the AG system post‐intestinal fluid addition. Over 30 min of digestion, particle count dwindled, possibly influenced by bile salts, pancreatic lipase, and small intestine ions collectively disrupting particle structures, progressively releasing the oil phase.

### 3.6 FFA Release

The choice of distinct emulsifying agents wielded influence over the physical and chemical characteristics of Pickering emulsions, thereby impacting lipid hydrolysis dynamics during simulated in vitro digestion. Throughout lipid digestion, the continual release of fatty acids precipitates a pH decline. Enzymatic hydrolysis kinetics were meticulously monitored by introducing NaOH to uphold the optimal pH level within the hydrolysis medium. Figure 9E illustrates the ultimate free fatty acid (FFA) release rates for EPGP, AG, and MG emulsions, tallying 77.56%, 59.25%, and 37.92%, respectively.

Research has underlined a correlation between FFA release rates from emulsions and the particle size of emulsion droplets. Fat degradation in the small intestine transpires via a chemical interfacial reaction. Smaller droplet sizes escalate specific surface area, fostering heightened interaction between oil droplets and digestive lipase, thus enhancing digestion efficiency and augmenting FFA release rates (Costa et al. 2020; Wijaya et al. 2020). Notably, the pronouncedly elevated FFA release rate of EPGP compared to AG and MG may be ascribed to its diminutive particle size. EPGP emulsion exemplified rapid release rates within the first 30 min of digestion, plateauing after 80 min, indicative of comprehensive lipid digestion.

## 4 Conclusion

A narrow, symmetrical peak of homogeneous EPGP, boasting a molecular weight of 1.3 × 10^6^ Da, was successfully derived from peach gum via xylanase extraction. This method of extraction notably reduces the molecular weight relative to alternative techniques. The β‐glycosidic bond linkage of polysaccharides and the broad variation in monosaccharide composition were elucidated. The scavenging potential of EPGP against DPPH, OH, ABTS cation, and O^2˗^ free radicals surpassed that of WPGP, underscoring the heightened antioxidant efficacy attributed to low molecular weight PGP. In contrast to AG and MG, the EPGP emulsion showcased superior emulsification properties, heightened resistance to digestion, and a heightened FFA release rate, offering potential enhancements in the efficiency of lipid‐soluble active compound utilization. The proposed structure is consistent with NMR data, though exact branching patterns require further verification. Low molecular weight peach gum polysaccharides exhibit enhanced antioxidant activity and improved emulsifying properties to some extent, but whether they affect other bioactivities remains to be explored. Animal experiments could be conducted to validate the digestive characteristics of peach gum polysaccharides. Leveraging the emulsifying properties of peach gum polysaccharides, lipid‐soluble active substances could be loaded to achieve sustained release and improve the utilization efficiency of these bioactive compounds.

## Author Contributions

**Xiaohua Cheng:** conceptualization (lead), data curation (equal), funding acquisition (lead), project administration (equal), resources (equal), writing – original draft (equal). **Shubo Chen:** data curation (equal), methodology (lead), writing – original draft (equal). **Yang Liu:** formal analysis (lead). **Feng Yang:** software (lead), validation (lead). **Jie Chen:** project administration (equal), resources (equal), supervision (lead), writing – review and editing (lead).

## Conflicts of Interest

The authors declare no conflicts of interest.

## Data Availability

The data that support the findings of this study are available from the corresponding author upon reasonable request.
